# Two Decades of Endovascular Abdominal Aortic Aneurysm Repair: Enormous Progress With Serious Lessons Learned

**DOI:** 10.1161/JAHA.111.000075

**Published:** 2012-06-22

**Authors:** Andres Schanzer, Louis Messina

**Affiliations:** University of Massachusetts Medical SchoolWorcester, MA

**Keywords:** abdominal aortic aneurysm, endovascular repair, stent

The modern open surgical management of abdominal aortic aneurysm (AAA) has changed little since its inception in the 1950s. Endoaneurysmorrhaphy, first described by Rudolph Matas in 1888, involved ligating the branches of an aneurysm from within the aneurysm sac. Approximately 25 years later at the beginning of the 20th century, Alexis Carrel received the Nobel Prize for demonstrating the feasibility of suture repair of arteries and perfecting an anastomotic technique to join 2 vessels. With these techniques established, an AAA could be repaired by anastomosis of a synthetic conduit to the aorta just proximal and distal to the AAA, thereby preserving antegrade blood flow.^1^Dubost was the first to marry these 2 techniques in 1952, with the first report of a successful open AAA repair with homograft replacement.^2^ Aside from the development of various different types of conduit materials, open AAA repair has remained largely unchanged through to the present day.

The most dramatic shift in the surgical management of AAA occurred in 1991, when Juan Parodi reported the first endovascular AAA repair (EVAR).^3^ This transformative moment marks the beginning of minimally invasive AAA repair as an alternative to open surgical repair. Whereas the elective management of AAA traditionally had depended solely on open surgical repair,^4,5^ these recent developments in catheter-based, endovascular techniques led to a substantial increase in the proportion of AAAs managed electively with EVAR. In 2006, only 15 years after the initial EVAR report, 21 725 EVAR procedures were performed in the United States, exceeding for the first time the number of open surgical AAA repairs.^6^ Currently, >70% of elective AAA repairs in the United States are performed with EVAR.^6^

Although the goals of EVAR and open surgical AAA repair are identical (ie, to eliminate the risk of death from rupture of AAA), the treatment strategy underlying EVAR is completely different than that of open surgical repair. During open repair, the aorta and iliac arteries are clamped, thereby increasing aortic resistance and inducing pelvic and lower-extremity ischemia; the aneurysm is opened; branch vessels are suture-ligated; the aortic aneurysm is replaced with a prosthetic graft; clamps are removed; and blood flow is restored to the pelvis and lower extremities. During EVAR, the aneurysm is left intact, but all blood flow is excluded from the aneurysm by catheter-based deployment of a stent graft, without the necessity to transiently occlude the aorta (Figure 1).

**Figure 1.**
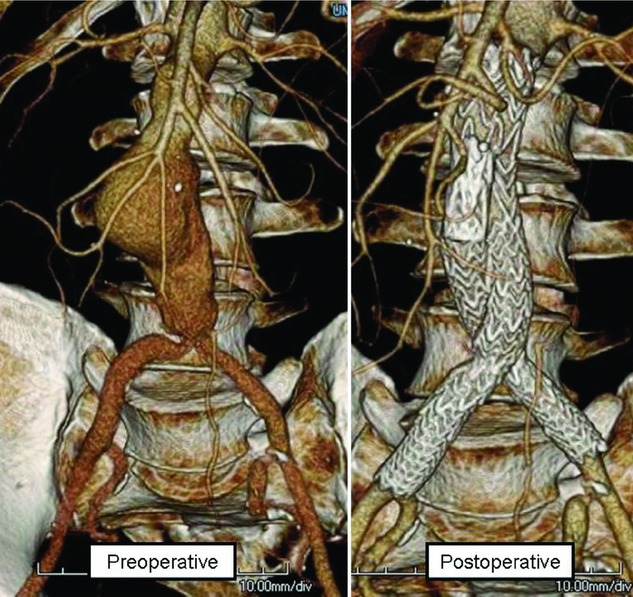
Endovascular repair of an infrarenal AAA demonstrating the preoperative and postoperative anatomy on CT scan with 3-dimensional reconstruction.

Just as the treatment strategy underlying EVAR is entirely different from that of open AAA repair, the modes of failure also are entirely different. Because the AAA is left intact after EVAR, the patient remains at risk of AAA rupture should flow to the aortic aneurysm sac persist. This phenomenon of persistent flow into an aortic aneurysm sac, despite stent graft placement, is called an endoleak. Endoleaks have been categorized into 4 subgroups on the basis of the location of their inflow into the aortic aneurysm sac (Figure 2). A type I endoleak is caused by persistent flow into the aneurysm sac through either the proximal or the distal endograft attachment site as a result of failure to achieve a complete seal between the endograft and the aortic or iliac artery wall. A type II endoleak is caused by retrograde flow into the aneurysm sac, usually from either a lumbar artery or the inferior mesenteric artery. A type III endoleak is caused by flow into the aneurysm sac that is due to an inadequate overlap at a junction of modular graft components or to a defect in the graft fabric. A type IV endoleak is caused by blood flow through the pores in the fabric of the stent graft into the aneurysm sac.

**Figure 2.**
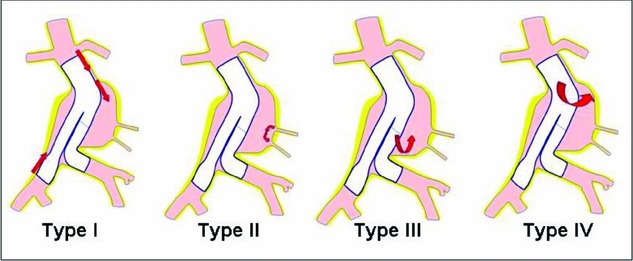
Types of endoleak.

Three large, prospective, randomized controlled trials have compared outcomes after elective open AAA repair to those after EVAR (Table 1).^7–9^ In general, the findings across all 3 studies have been concordant.

**Table 1.** Summary of the Results From the Randomized Controlled Trials and the Medicare Propensity Score–Matched Comparison of EVAR With Open AAA Repair

Trial	Short-Term Death Rate*	Long-Term Death Rate†
EVAR 1 Trial^7^		

EVAR (n=626)	1.8% at 30 d	23.1% at 4 y

Open AAA (n=626)	4.3% at 30 d	22.3% at 4 y

DREAM Trial^8^		

EVAR (n=173)	1.2% at 30 d	31.1% at 6 y

Open AAA (n=178)	4.6% at 30 d	30.1% at 6 y

OVER Trial^9^		

EVAR (n=444)	0.5% at 30 d	7.0% at 2 y

Open AAA (n=437)	3.0% at 30 d	9.8% at 2 y

Endovascular vs. Open Repair of Abdominal Aortic Aneurysms in the		

Medicare Population^10^		

EVAR (n=22 830)	1.2% at 30 d	34.0% at 5 y

Open AAA (n=22 830)	4.8% at 30 d	34.3% at 5 y

* *P*<0.05 for each comparison in each trial.

† *P* nonsignificant for each comparison in each trial.

## Randomized Clinical Trials Comparing Outcomes After EVAR and Open AAA Repair

### The EVAR 1 Trial

The United Kingdom Endovascular Aneurysm Repair (EVAR) 1 trial^7^ enrolled patients between 1999 and 2004 from 37 hospitals in the United Kingdom. Twelve hundred fifty-two patients whose maximum aortic aneurysm diameter was >5.5 cm and who were deemed to have an acceptable risk of postoperative death for both procedures were randomized to undergo either open or endovascular repair. Patients were followed up for rates of perioperative and late death, graft-related complications, reinterventions, and resource use until the end of 2009.

The 30-day immediate postoperative death rates were 1.8% after EVAR and 4.3% after open repair (*P*=0.02), a remarkable advantage for EVAR. However, to the surprise of many, at the end of follow-up, both all-cause death rates (survival curve convergence at 2 years) and aneurysm-related death rates (survival curve convergence at 6 years) were equivalent between these 2 groups. The rates of graft-related complications and reinterventions were higher after EVAR, thereby resulting in higher overall costs for EVAR.

### The DREAM Trial

The Dutch Randomised Endovascular Aneurysm Management (DREAM) Trial^8^ enrolled patients between 2000 and 2003 from 26 centers in the Netherlands and 4 centers in Belgium. Three hundred fifty-one patients whose maximum AAA diameter was >5.0 cm and who were deemed to have acceptable risk for both procedures were randomized to undergo either open or endovascular repair. The primary study outcomes were rates of death from any cause and reintervention. Patients were followed up through 2009.

The 30-day postoperative death rates were 1.2% after EVAR and 4.6% after open repair (risk ratio 3.9; 95% confidence interval, 0.9 to 32.9). By the end of follow-up, all-cause death rates (survival curve convergence at 1 year) were equivalent between groups. The rates of graft-related complications and reinterventions were higher after EVAR.

### The OVER Trial

The Open Versus Endovascular Repair (OVER) trial^9^ enrolled patients between 2002 and 2008 from 42 Veterans Affairs Medical Centers in the United States. Eight hundred eighty-one patients whose AAA maximum diameter was >5.0 cm, iliac aneurysm was >3.0 cm, or AAA was >4.5 cm and who had rapid AAA enlargement (>0.5 mm in 6 months) or saccular morphology, who were deemed to have suitable risk for both procedures, were randomized to undergo either open or endovascular repair. The only report to date from this trial represents interim data analysis, at which point the mean follow-up duration of randomized patients was 1.8 years. The primary study outcomes are procedure failure, secondary therapeutic procedures, length of stay, quality of life, erectile dysfunction, major morbidity, and death.

The 30-day death rates were 0.5% after EVAR and 3.0% after open repair (*P*=0.004). By the end of follow-up, all-cause death rates (survival curve convergence at 2 years) were equivalent between groups. Patients in the EVAR group had reduced procedure time, blood loss, transfusion requirement, duration of mechanical ventilation, length of hospital stay, and length of intensive care unit stay. There were no differences between the 2 groups in major morbidity, procedure failure, secondary therapeutic procedures, aneurysm-related hospitalizations, health-related quality of life, or erectile dysfunction.

### Trial Results

The results from these 3 prospective randomized trials that compared early and late outcomes after open and endovascular repair of AAA were remarkably consistent in all major respects. In aggregate, the findings can be summarized as follows: (1) The perioperative morbidity and mortality rates are significantly lower after EVAR than after open repair of AAA. (2) The short-term survival advantage of EVAR diminishes during long-term follow-up, such that if patients survive beyond ≈2 years, the long-term survival rates of patients are similar in both groups. (3) Although the reintervention rate after EVAR is higher than after open repair, most of these reinterventions are performed with catheter-based techniques, albeit at overall higher costs.

To better understand how these randomized trial findings may generalize to the US population as a whole, Dr Schermerhorn and colleagues compared short-term survival, long-term survival, and reinterventions after EVAR and open AAA repair in propensity score–matched cohorts of Medicare beneficiaries undergoing repair during the 2001–2004 period, with follow-up through 2005.^10^ This analysis of 22 830 matched patients yielded results consistent with the aforementioned randomized trials. The 30-day death rates were 1.2% after EVAR and 4.8% after open repair (*P*<0.0001). By the end of follow-up, all-cause death rates (survival curve convergence at 3 years) were equivalent between groups. Reinterventions related to AAA repair were more frequent in patients who underwent EVAR (9.0% versus 1.7%, *P*<0.0001). Interestingly, the authors also used procedure codes to calculate “surgery for laparotomy-related complications” and found that these occurred more frequently after open repair of AAAs (9.7% versus 4.1%, *P*<0.0001).

### A Note of Caution

Although each study shows that EVAR confers a substantial early survival benefit, this benefit is lost in every trial at different points of long-term follow-up. Despite being the subject of numerous studies, consensus around a satisfactory explanation for this conundrum has not emerged. Possible explanations include poor compliance with follow-up resulting from patients not fully understanding the critical importance of lifetime surveillance, device fatigue and subsequent failure, persistence of an elevated inflammatory state associated with the presence of an intact aneurysm leading to cardiovascular events, and device failures due to treatment of unfavorable anatomy outside of the specified Instructions for Use (IFU).

Rates of AAA sac enlargement after EVAR are not negligible. In a large university series, Hogg and colleagues^11^ demonstrated the rate of aortic sac enlargement after EVAR to be 21% at 5 years. In another recent study, even in patients treated for a type II endoleak in whom surveillance detected AAA sac enlargement, 55% continued to show expansion >5 mm by 5 years after treatment.^12^

To appropriately interpret the studies that compare outcomes after EVAR and open AAA repair, it is important to understand the context in which they were obtained. The clinical trials for regulatory approval and postmarketing analyses, as well as the randomized controlled trials that compared EVAR to open AAA repair, have included only patients in whom the specific anatomic requirements defined in the device IFU were met.^9,13–16^ Studies utilizing national databases also have been limited in that these studies lack access to preoperative and postoperative aortic and iliac artery anatomic measurements and therefore have been unable to assess whether devices were used in accordance with published IFU or whether adherence to IFU affected the rate of device failure and therefore clinical outcomes.^6,10^ Thus, the proportion of patients and the outcomes of patients who undergo EVAR with anatomy outside of the device IFU are largely undocumented with regard to short- and long-term death and complication rates, with the exception of a small number of single-center reports.^17–19^

The issue of adherence to the specific anatomic requirements defined in the device IFU is of paramount importance when the long-term results of the EVAR trial mentioned above are considered.^7^ The late follow-up of this cohort demonstrated that the early survival advantage of patients undergoing EVAR disappeared with time and that a significant proportion of late deaths after EVAR were due to aneurysm rupture (27 AAA ruptures in the EVAR group; 5 within 30 days of surgery and 22 after that).^7^ Although the exact mechanism was not determined for each patient who died because of aortic rupture after EVAR in the EVAR study, the authors thought that the early aortic ruptures would have been preventable had a computed tomography (CT) scan been done before discharge.^20^ The cases of late aortic rupture and death were found to be closely linked to aortic aneurysm sac enlargement.^20^ Because aortic rupture has been shown to be an important cause of late death in highly selected patient populations within clinical trials, it is reasonable to hypothesize that commercial use of EVAR devices in patients who did not meet device IFU could result in a higher rate of postoperative aortic sac enlargement and thereby put such patients at higher risk of aortic rupture.

## IFU Compliance Study

To address the rate of compliance with IFU, we conducted a study on data from a large, multicenter cohort CT scan database to determine the degree of compliance with IFU anatomic guidelines for EVAR, to examine changes in compliance with the IFU over the past decade, and to determine the relationship between baseline aortic and iliac artery anatomic characteristics and incidence of aortic aneurysm sac enlargement after EVAR.^21^ The primary limitation of this study was that although the number of patients studied was large, no clinical characteristics of the patients were available, and the generalizablilty of this population to patients undergoing EVAR in the United States could not be established. Similarly, no information was available about which, if any, interventions were performed in response to the findings of a CT scan.

Patients undergoing EVAR between January 1, 1999, and December 31, 2008, were assembled from a medical imaging repository at M2S, Inc. (West Lebanon, NH). Utilizing standardized algorithms, M2S creates three-dimensional computer models from CT images of aortic aneurysms. In addition to serving as the core imaging lab for several large aneurysm management trials,^22–24^ M2S also provides these services to both private and academic hospitals throughout the world. For purposes of this study, M2S provided de-identified data on all patients in their prospectively acquired database who underwent a CT scan before EVAR and had at least 1 CT scan after EVAR between 1999 and 2008 in the United States. On the basis of these criteria, 10 228 patients in the United States who underwent EVAR for AAA repair between 1999 and 2008 were identified.

**Figure 3.**
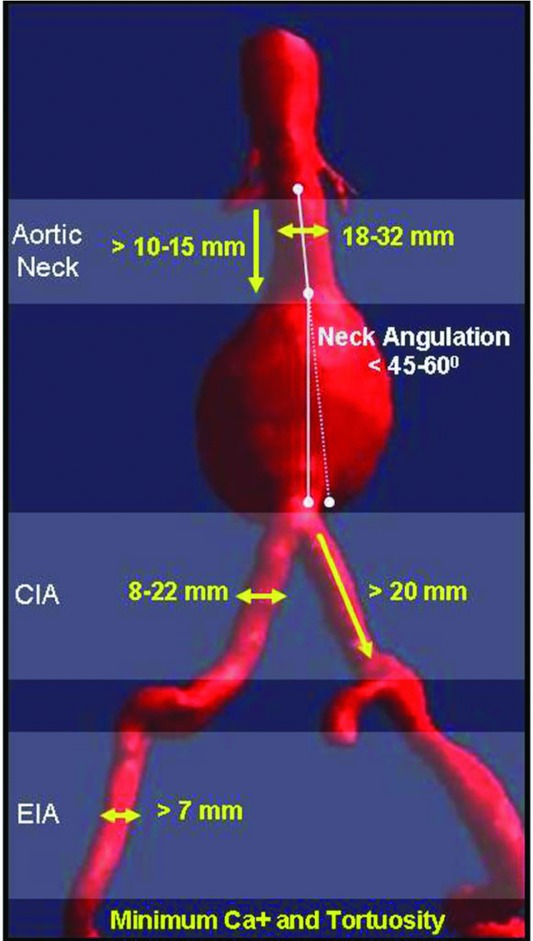
The aortic and iliac arterial anatomy boundary conditions defined by the IFU that are packaged with each FDA-approved commercial endovascular aortic device. CIA indicates common iliac artery; EIA, external iliac artery .

This study demonstrated that in this cohort of patients the incidence of AAA sac enlargement after EVAR was 41% at 5 years, a rate that increased over the time period of the study. When all EVAR-treated patients were classified according to IFU criteria, 5983 (58.5%) patients were outside of compliance with the most conservative device IFU available on the market, and 3178 (31.1%) patients were outside of the most liberal IFU available on the US market (Figure 3). Liberalization of the anatomic characteristics deemed suitable for EVAR has occurred, and several of these factors, including aortic neck diameter, aortic neck angle, and common iliac artery diameter, were independently associated with aortic aneurysm sac enlargement (Table 2). These observations raise the question of whether such liberalization is justified for current endovascular device designs.

**Table 2.** Significant Independent Predictors for Aortic Aneurysm Sac Enlargement Identified on Multivariable Cox Proportional-Hazards Analysis

Covariates	Hazard Ratio (95% Confidence Interval)	*P*
Age, y		

<60	REFERENCE	

60–69	0.80 (0.60–1.05)	0.11

70–79	0.87 (0.67–1.14)	0.31

≥80	1.32 (1.03–1.75)	0.05

Female	0.96 (0.82–1.13)	0.64

AAA diameter		

Maximum AAA diameter ≥55 mm	0.97 (0.86–1.10)	0.62

Aortic neck length, mm		

>15	REFERENCE	

10–15	0.87 (0.71–1.07)	0.19

<10	0.94 (0.77–1.15)	0.53

Aortic neck diameter at lowest renal artery, mm		

<28	REFERENCE	

28–32	1.80 (1.44–2.23)	<0.0001

>32	2.07 (1.46–2.92)	<0.0001

Conical neck	1.17 (0.97–1.42)	0.10

Aortic neck Angle		

<45°	REFERENCE	

45–60°	1.04 (0.90–1.21)	0.58

>60°	1.96 (1.63–2.37)	<0.0001

Iliac diameter		

Both common iliac arteries ≤20 mm	REFERENCE	

Only 1 common iliac artery >20 mm	1.46 (1.21–1.76)	<0.0001

Both common iliac arteries >20 mm	1.31 (0.99–1.74)	0.06

Endoleak during follow-up	2.70 (2.40–3.04)	<0.0001

This analysis of M2S data was meant to be a starting point for a critical conversation in the evolving field of endovascular aneurysm repair, rather than a conclusion. It now has been established unambiguously that the risk of late rupture after EVAR is higher than initially believed.^20^ A consensus exists that the primary anatomic determinant of late AAA rupture after EVAR is aortic sac enlargement.^20,25^ It is likely that the rate of aortic sac enlargement after EVAR will be dependent on the specific patient population and endovascular device studied. For example, the aforementioned recent study by Hogg et al^11^ found the rate of aortic sac enlargement after EVAR to be 21% at 5 years; although less than the 41% found in this study, this rate of late aortic sac enlargement is still far higher than previously documented. According to this analysis of patients undergoing EVAR in the M2S database, patients undergo EVAR outside industry-recommended guidelines frequently, and this practice increases the risk of late aortic sac enlargement.

## The Future

Undoubtedly, EVAR represents a tremendous advance in the treatment of AAA and has provided significant benefit to many patients. Nevertheless, if the widespread application of this technique continues to grow in patients with unfavorable anatomy, these benefits will be offset by increased rates of treatment failure, costly reinterventions, and the potential for late aneurysm rupture. In this context, we are reminded that the high rate of technical feasibility and the outstanding short-term results after EVAR are meaningful to patients only if the ability of EVAR to protect them against death due to AAA rupture is durable over the long term.

A prospective EVAR registry that incorporates an independent imaging registry is necessary to define more precisely the specific aortic and iliac artery anatomic characteristics suitable for EVAR with currently available commercial devices. In patients with anatomy proven to be disadvantaged for currently available commercial devices, endovascular technologies need to evolve so that these anatomic challenges can be treated more effectively. Although the next-generation EVAR devices, the branched and fenestrated endovascular grafts available at some select sites in trials,^26–28^ do provide a means to treat patients with anatomy unfavorable for standard EVAR, these devices are still limited by complexity, requirement for large doses of radiation, prolonged procedure times, expense, and availability. Finally, it is imperative that vascular surgery training programs remain committed to maintaining the technical skills of their trainees to perform open surgical repair of AAAs that do not have anatomy amenable to EVAR. Creative training solutions that incorporate realistic simulation with tactile feedback may be necessary as the number of open aortic cases continues to decline.

In summary, over the past 2 decades, vascular surgeons have successfully introduced and embraced a new, minimally invasive approach to the treatment of AAA. Countless patients have benefitted from EVAR, and it should be noted that in an exceptionally brief span of time, vascular surgeons have developed and implemented the necessary skill set required to provide EVAR to patients safely, with extremely low risk of perioperative death. We now find ourselves at a critical moment that requires a rigorous assessment of the advantages and disadvantages of EVAR, as it has become the mainstay for AAA treatment. Device development with a focus on durability to prevent late AAA sac enlargement and rupture is an imperative. Although next-generation EVAR devices, such as the highly promising branched and fenestrated solutions currently undergoing investigation, will expand the anatomic boundary conditions suitable for successful EVAR, current technology makes careful patient selection crucial. Caution should be exercised when patients selected for EVAR do not meet device IFU, and, most importantly, each patient and treating physician must commit to life-long follow-up incorporating careful endograft imaging surveillance.
